# Interactions of Tumor Necrosis Factor–Related Apoptosis-Inducing Ligand (TRAIL) with the Immune System: Implications for Inflammation and Cancer

**DOI:** 10.3390/cancers11081161

**Published:** 2019-08-13

**Authors:** Katharina Beyer, Ann-Kathrin Baukloh, Ani Stoyanova, Carsten Kamphues, Arne Sattler, Katja Kotsch

**Affiliations:** Charité-Universitätsmedizin Berlin, Department of General, Visceral and Vascular Surgery, Campus Benjamin Franklin, Hindenburgdamm 30, 12203 Berlin, Germany

**Keywords:** TRAIL, apoptosis, cancer, inflammation, immune system

## Abstract

Tumor necrosis factor (TNF)-related apoptosis-inducing ligand (TRAIL) is a member of the TNF superfamily. TRAIL has historically been distinct from the Fas ligand and TNFα in terms of selective apoptosis induction in tumor cells and has a nearly non-existent systemic toxicity. Consequently, in the search for an ideal drug for tumor therapy, TRAIL rapidly drew interest, promising effective tumor control with minimal side effects. However, euphoria gave way to disillusionment as it turned out that carcinoma cells possess or can acquire resistance to TRAIL-induced apoptosis. Additionally, studies on models of inflammation and autoimmunity revealed that TRAIL can influence immune cells in many different ways. While TRAIL was initially found to be an important player in tumor defense by natural killer cells or cytotoxic T cells, additional effects of TRAIL on regulatory T cells and effector T cells, as well as on neutrophilic granulocytes and antigen-presenting cells, became focuses of interest. The tumor-promoting effects of these interactions become particularly important for consideration in cases where tumors are resistant to TRAIL-induced apoptosis. Consequently, murine models have shown that TRAIL can impair the tumor microenvironment toward a more immunosuppressive type, thereby promoting tumor growth. This review summarizes the current state of knowledge on TRAIL’s interactions with the immune system in the context of cancer.

## 1. Introduction

Cancer is a global burden, and according to current projections, one in eight men and one in 11 women will die from cancer [1]. In the search for an ideal drug for tumor therapy, tumor necrosis factor (TNF)-related apoptosis-inducing ligand (TRAIL) has drawn interest, as it was considered to selectively induce apoptosis in tumor cells, thereby not affecting healthy body cells [2]. These properties suggested TRAIL was a promising drug for cancer therapy. However, preclinical data could not be translated into clinical success. One reason was that carcinoma cells possess or can acquire resistance to TRAIL-induced apoptosis. Furthermore, TRAIL can significantly affect the immune system and thus alter the success of tumor therapy. This review summarizes current knowledge on the interaction of TRAIL with the immune system, and specifically discusses the influence of TRAIL on the tumor microenvironment (TME).

## 2. Discovery of TRAIL

The balance of self-renewal and cell death is an important feature of healthy tissue. A disturbance of this balance through uncontrolled cell proliferation, as well as resistance toward apoptosis, is an important hallmark of carcinogenesis and tumor progression. Chemotherapeutics can inhibit cell division. However, the selectivity of the effect is only determined by the rate of cell division, resulting in a broad spectrum of side effects. The selective induction of apoptosis in tumor cells, though, appears to be a much more targeted strategy. Apoptosis can occur by binding ligands of the TNF superfamily to corresponding death receptors. However, TNFa and FasL, both members of the TNF superfamily, were found to be extremely toxic, making clinical use problematic [3,4]. In the mid-nineties, Wiley and Pitti independently discovered a new member of the TNF superfamily, which was called TRAIL or Apo 2-ligand [5,6]. Subsequently, two properties were identified that make TRAIL unique compared to the other members of the TNF superfamily: first, it mainly induces apoptosis in transformed cells and does not significantly affect healthy cells; second, TRAIL messenger RNA is also found around healthy tissue, while expression of the remaining members of the TNF superfamily is tightly regulated and often transient [7]. The latter suggests a physiological significance of TRAIL beyond tumor defense.

## 3. The TRAIL Receptor System

**Figure 1:**
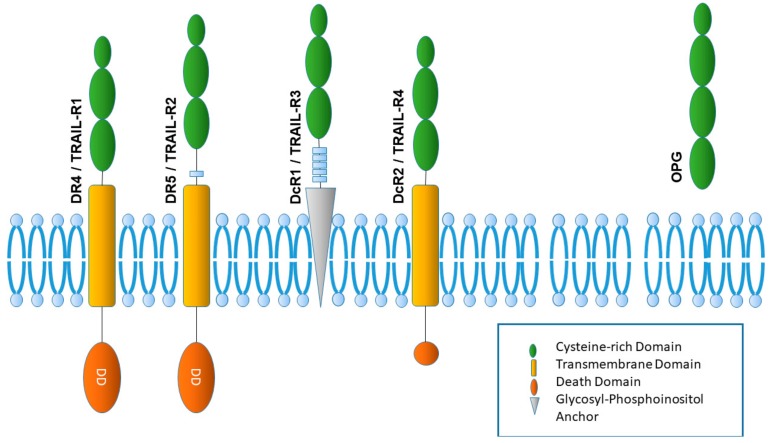
The human TRAIL receptor system. In humans, two TRAIL receptors are known to possess a functional death domain and thus can induce apoptosis: TRAIL receptor 1 (TRAIL-R1, DR4) and TRAIL receptor 2 (TRAIL-R2, DR5). Besides these are two other membrane-bound TRAIL receptors serving as decoy receptors: TRAIL receptor 3 (decoy receptor 1, DcR1) has no intracellular domain, while TRAIL receptor 4 (DcR2) contains a truncated death domain. In addition to the four membrane-bound TRAIL receptors, there is a soluble receptor known as osteoprotegerin (OPG).

In humans, two TRAIL receptors are currently known to possess a functional death domain and thus can induce apoptosis: TRAIL receptor 1 (TRAIL-R1, DR4) and TRAIL receptor 2 (TRAIL-R2, DR5), which were discovered in the late nineties [8,9,10] (Figure 1). In mice, only one TRAIL receptor bearing a functional death domain exists: DR5 (TRAIL-R2), which is not structurally identical to either of the two human DRs [11]. TRAIL-R1/TRAIL-R2 trimerization on TRAIL binding can lead to the assembly of the death-inducing signaling complex (DISC) platform at their cytoplasmatic domain. The DISC complex is composed of trimerized TRAIL receptors and the Fas-associated death domain protein (FADD), which can use its death effector domain (DED) to recruit proteins, such as procaspase 8 and procaspase 10, as well as cellular FLICE (FADD-like IL-1β-converting enzyme)-inhibitory protein cellular FLICE (FADD-like IL-1β-converting enzyme)-inhibitory protein (cFLIP) long (cFLIP_L_) and short (cFLIP_s_) isoforms. Eventually, this can lead to procaspase 8/10-dimerization, finally resulting in the activation of downstream caspases.

**Figure 2:**
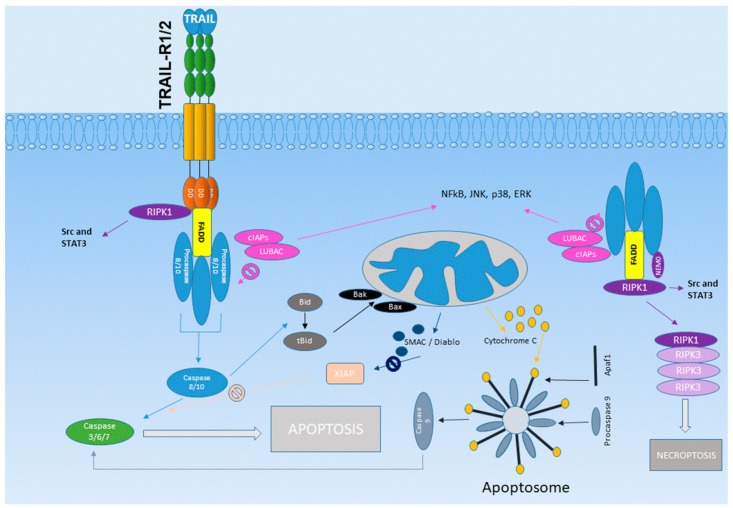
TRAIL-R1/R2 signaling. TRAIL-R1/TRAIL-R2 trimerization upon TRAIL-binding can lead to death-inducing signaling complex (DISC) formation, which is composed of trimerized TRAIL receptors and Fas-associated death domain protein (FADD), which in turn can use its death effector domain (DED) domain to recruit proteins, such as procaspase 8 and procaspase 10, as well as cFLIP isoforms. Depending on the cell type, activation of the DISC may lead to complete activation of the caspase cascade, or may require mitochondrial proteins, which in turn antagonize XIAP. Cellular inhibitors of apoptosis proteins 1/2 (cIAPs) can lead to ubiquitylation of RIPK1 and thus to the recruitment of LUBAC, which alleviates the activation of caspase 8. RIPK1 is obligatory for the stimulation of Src and STAT3. Additionally, the binding of TRAIL to TRAIL-R1/2 can induce the formation of a second cytosolic complex through which necroptosis can be induced when the activation of caspases is inhibited. Both TRAIL-induced signaling complexes can activate NF-κB, p38 mitogen-activated protein kinase, JUN N-terminal kinase (JNK), and extracellular signal-regulated kinase (ERK), and can thus promote tumor growth and invasiveness. Abbreviations: TRAIL (TNF-related apoptosis inducing ligand); DISC (death-inducing signaling complex); FADD (Fas-associated death domain-domain protein); DED (death effector domain); cFLIP (cellular FLICE (FADD-like IL-1β-converting enzyme)-inhibitory protein); XIAP (X-linked inhibitor of apoptosis protein); LUBAC (linear ubiquitin chain assembly complex); RIPK (receptor-interacting serine/threonine-protein kinase); Src (tyrosine-protein kinase Rous sarcoma oncogene cellular homolog); STAT (signal transducer and activator of transcription); JNK (JUNN-terminal kinase); ERK (extracellular signal-regulated kinase).

A paradigm shift has recently taken place regarding the role of cFLIP. In a recent work, Hughes et al. [12] showed that cFLIP does not compete with caspase 8, but that caspase cooperatively recruits and heterodimerizes with cFLIP. The composition of the caspase 8:cFLIP_L/S_ heterodimer is decisive for whether downstream caspases are activated. Procaspase 8:cFLIP_L_ acts preferentially as an activator, promoting DED-mediated procaspase 8 oligomer assembly, whereas procaspase 8:cFLIP_S_ lacks activity and potently blocks procaspase 8 activation [12]. Depending on the cell type, activation of the DISC may lead to complete activation of the caspase cascade, or may require mitochondrial proteins, which in turn antagonize the X-linked inhibitor of apoptosis protein (XIAP), an inhibitor of caspases 3, 7, and 9. This pathway represents a cross-linking between extrinsic and intrinsic apoptosis induction [13] (Figure 2).

Next to the abovementioned receptors with a functional death domain, other TRAIL receptors serve as decoy receptors: TRAIL receptor 3 (decoy receptor 1, DcR1) has no intracellular domain [8,14], while the death domain for TRAIL receptor 4 (DcR2) contains a truncated death domain [15,16,17]. These receptors have been believed to compete for TRAIL binding, thus impairing the apoptotic potential of TRAIL [18]. However, this possible competitive effect of decoy receptors requires a correspondingly high level of expression of these receptors compared to the other receptors with similar binding affinity. The fact that the binding affinity is the same, and the expression of the decoy receptors is not higher in many healthy human cells, led to the hypothesis that other mechanisms have to be more determinant of whether a cell is TRAIL-resistant or not [19]. Nevertheless, the binding of TRAIL to TRAIL-R4 can activate nuclear factor (NF)-kB, which in turn leads to synthesis of anti-apoptotic proteins [15]. The detailed signal transduction pathway of these receptors for the initiation of cell death is not well understood, as both the overexpression of decoy receptors and the formation of inactive heterocomplexes with TRAIL-R2 and TRAIL-R4 are associated with impaired TRAIL-induced apoptosis [20].

In addition to the four membrane-bound TRAIL receptors, there is a soluble receptor known as osteoprotegerin (OPG) [21]. While application of OPG may lead to the inhibition of TRAIL-induced apoptosis, this effect seems to be insignificant under physiological conditions due to the lower binding affinity between TRAIL and OPG [22].

Where inhibition of caspase 8 leads to the failure of apoptosis induction, TRAIL stimulation may lead to necroptosis under certain conditions. This pathway is mainly dependent on the expression of the receptor-interacting serine/threonine-protein kinase 3 (RIPK3) [23].

Separately from the classical signal transduction pathways via caspase 8 activation leading to apoptosis, TRAIL can activate alternative signal transduction pathways, provoking NF-kB, mitogen-activated protein kinase (MAPK), Src, and Phosphatidylinositol-3-Kinase (PI3K), which are known to promote the malignancy of carcinoma cells [24]. Activation of NF-kB leads to the increased formation of anti-apoptotic proteins, which in turn reduces TRAIL sensitivity of the cell [25].

The mechanisms that determine whether a cell becomes apoptotic through TRAIL stimulation are not yet fully understood. Nevertheless, several mechanisms have been discovered in recent decades that allow cells to influence their TRAIL sensitivity. To detail these mechanisms is beyond the scope of this review. In brief, additional to the ratio of decoy receptors to death receptors, the posttranslational modification of death receptors plays a role in TRAIL sensitivity [26]. As well as the receptors, the ratio of pro- to anti-apoptotic intracellular proteins is decisive. Therefore, an inhibitor of apoptosis proteins is particularly crucial [7]. These proteins can in turn be synthesized by activation of NF-kB.

## 4. Clinical Development of TRAIL Agonists

The selective induction of apoptosis in tumor cells makes TRAIL an ideal drug in tumor therapy. Accordingly, various preclinical and clinical studies on the efficacy of TRAIL and TRAIL receptor antibodies have been performed and are still in progress. A detailed summary of these studies is beyond the scope of this review and has been conducted elsewhere [13]. In summary, though, recombinant soluble TRAIL (AMG 951, dulanermin) showed excellent tolerability in phase I studies [27,28]. However, effects were only marginal in a phase III study [27]. The main problems were the short half-life of dulanermin and its suboptimal apoptotic effect. Possibilities that have been investigated to improve the apoptotic potential are the fusion of TRAIL with the Fc portion of human immunoglobulin (Ig) G1 and the production of hexavalent TRAIL receptor antagonists [13]. In addition, agonistic antibodies against the TRAIL receptor 1 and the TRAIL receptor 2 have been developed, which also showed good tolerability with a suboptimal apoptotic effect [29].

Central to the further development of these therapeutic approaches is the enhancement of the TRAIL sensitivity of tumor cells, as well as the development of TRAIL receptor agonists with high apoptotic potential. Whether these strategies will help TRAIL to achieve the initially anticipated success in tumor therapy remains to be seen.

## 5. Tumor-Promoting Side Effects of TRAIL

Apart from inducing apoptosis, interactions of TRAIL with tumor cells can initiate their migration and invasion [30], thereby promoting tumor growth and metastasis. These tumor-promoting properties of TRAIL have been demonstrated in cell lines, as well as in murine models. For instance, the binding of TRAIL by pancreatic carcinoma cell lines can lead to apoptosis induction, as well as NF-kB activation, which in turn protects cells from TRAIL-induced apoptosis [31]. TRAIL-induced NF-kB activation also results in the release of proinflammatory cytokines by pancreatic cancer cell lines, including interleukin (IL)-8, monocyte chemoattractant protein 1 (MCP1), and the urokinase-type plasminogen activator (uPA) [32]. Retroviral transfection of the pancreatic carcinoma cell line Colo357 with B-cell lymphoma-extra large (Bcl-xL) resulted in resistance toward TRAIL, leading to increased proinflammatory cytokine production. Production of cytokines was also detected in the TRAIL-sensitive cell line Colo357, albeit less than in the transfected TRAIL-resistant Colo357/Bcl-xL. Furthermore, TRAIL-treatment enhanced the invasive potential of Colo357/Bcl-xL cells, and this effect was dependent on uPA.

Most importantly, the administration of TRAIL to tumor-bearing mice led to increased metastasis in an orthotopic xenograft pancreatic carcinoma model employing severe combined immunodeficient(SCID) mice [32]. The fact that SCID mice have to be considered immune-deficient indicates that the increased metastasis and proliferation with TRAIL therapy is a direct effect of TRAIL on tumor cells, rather than TRAIL stimulation on the immune system. In concordance with these results, TRAIL stimulated the invasion of colorectal tumor cells, as well as liver metastases in a Kirsten Rat Sarcoma (KRAS)-dependent fashion. The loss of mutant KRAS abrogated this effect and promoted TRAIL-induced apoptosis [33].

In addition to the adverse effects mediated by TRAIL, the question was raised regarding why cancer cells retain their TRAIL-R expression when they are resistant to apoptosis induction by TRAIL. For instance, the clinical observation that increased the expression of DR5 implies a worse prognosis has led to the hypothesis that the expression of DR5 supports apoptosis-resistant tumors. This has been supported by results from cell culture experiments showing that the knockdown of DR5 leads to decreased proliferation of KRAS-mutated cell lines. These results have been confirmed in murine models showing that the expression of DR5 on cancer cells induces KRAS-driven tumor progression, invasion, and metastasis. This suggests that the effect is based on interactions between DR5 and tumor-owned TRAIL, without any participation from additional immune cells [34].

While TRAIL’s interactions with membrane-bound receptors have been examined in previous publications, recent studies have shown that TRAIL receptors are also present in the cytoplasm and nucleus of cancer cells. Nuclear DR5 has been found in higher amounts in cancer cells compared to non-transformed tissue, suggesting an important role of nuclear DR5 in tumor progression. In this context, Haselmann et al. recently described the nuclear expression of DR5 leading to increased tumor cell proliferation by inhibiting maturation of the microRNA let-7. Most importantly, this effect was not dependent on a receptor–ligand interaction with TRAIL [35]. In addition, tumor cells can translocate DR5 into autophagosomes, where it undergoes a lysosomal degradation, a mechanism that has recently been confirmed for circulating tumor cells leading to an increased resistance to apoptotic stimuli in these cells [36].

## 6. TRAIL: A Key Molecule for NK Cell-Mediated Immune Surveillance

As well as tumor cells, cells of the immune system can also express and secrete TRAIL on activation, indicating an additional important role of TRAIL for immune cell interactions [37,38,39,40]. TRAIL acts as a crucial element in cytotoxic effects mediated by cytotoxic T cells and natural killer (NK) cells [37,38], but other leukocytes including neutrophils, dendritic cells, and macrophages can also express TRAIL.

Cytotoxicity of NK cells is not only a prerequisite for the control of viral infections, but also for the efficient elimination of cancer cells, and therefore a hallmark for immune surveillance. In this process, recognition of tumor antigens leads to the activation of NK cells. According to the “missing self” hypothesis, NK cells can be activated when the target cell misses major histocompatibility complex (MHC) I expression. Cancer cells often downregulate MHC I expression to avoid detection by cytotoxic T cells, thereby becoming a target for NK cells [41]. In addition, activation receptors, such as Natural Killer Group 2D (NKG2D) recognize “induced-self” ligands, meaning ligands that are upregulated in malignant transformation, infection, or stress [42]. After ligation of their potential ligands, NK cells can kill tumor cells via the induction of apoptosis through the caspase pathway. In this context, as well as with the Fas ligand, TRAIL is a crucial effector molecule of NK cells [43]. In contrast to FasL, TRAIL can retain its apoptotic activity even in a soluble form that results from shedding of the membrane-bound TRAIL on NK cells [44]. Additional pathways of efficient target cell lysis are mediated via antibody-dependent, cell-mediated cytotoxicity and/or via direct lysis with perforin and granzyme. Recently, it has been shown that TRAIL expression on NK cells can be induced by activation of the intracellular receptor retinoic acid–inducible gene I (RIG-I) triggering enhanced cytotoxic killing [45].

Remarkably, murine NK cells freshly isolated from spleen, lung, and blood do not express TRAIL, whereas liver-resident CD3^−^DX5^+^ NK cells express TRAIL in low amounts. However, in vivo administration of IL-12 resulted in TRAIL^+^ interferon (IFN)γ^+^ NK cells that finally suppressed metastases in both liver and lung in a TRAIL-dependent fashion [46].

The observation that a subpopulation in the liver constitutively expresses TRAIL early led to the hypothesis that these cells are responsible for the defense against metastases. Mice deficient in IFNγ (IFNγ^−/−^) lack constitutively TRAIL-expressing NK cells in the liver, indicating that TRAIL expression on liver NK cells is dependent on IFNγ. The discovery that the addition of a neutralizing TRAIL antibody enhanced liver metastasis of TRAIL-sensitive tumors in mice, and that this effect did not occur in either IFNγ ^−/−^ or NK-depleted mice, supports the hypothesis that TRAIL-expressing NK cells in the liver are a natural defense against liver metastases [47,48].

In addition, a population of liver tissue-resident NK cells not requiring IL-2 or IL-15 for the induction of TRAIL expression has recently been categorized as type 1 innate lymphoid cells (ILC1s) [49]. ILC1s are defined by the production of IFNγ as the main cytokine, and the requirement of the transcription factor Tbet for development [50]. Thus, ILC1s include conventional NK cells, as well as intraepithelial ILC1s and helper ILC1s. In this context, ILC1s can carry out regulatory helper functions [51] that are attributed to TRAIL expression, which is in turn regulated by NKp46 [52]. These recent findings indicate that an in-depth understanding of the functions of ILC1s may lead to a better comprehension of the physiological functions of TRAIL.

## 7. TRAIL and Neutrophils: Lessons Learned from Models of Bacterial Infections

TRAIL was long thought to act selectively on tumor cells. This dogma has fallen apart in the last few years following various investigations involving inflammation models [53,54,55,56,57,58]. In this context, our group has demonstrated that administration of recombinant TRAIL increases the survival of diffuse peritonitis through a neutrophil-dependent mechanism applying a murine model of polymicrobial sepsis [53,54]. These findings are in line with results from other groups showing a protective role for TRAIL in zymosan-induced peritonitis, as well as in lipopolysaccharide-induced lung injury. In both models, the underlying mechanism was the induction of TRAIL-induced apoptosis in neutrophils [55] promoting the resolution of inflammation. In concordance, results from a murine model of *Streptococcus pneumoniae* pneumonia indicated a protective role of TRAIL. In this case, the proposed mechanism was apoptosis of alveolar macrophages induced by neutrophil-driven TRAIL [59].

In vitro data also showed that TRAIL could accelerate neutrophil apoptosis while its constitutive apoptosis was not regulated by TRAIL [60]. Interestingly, in murine peritonitis, the number of neutrophilic granulocytes in the peritoneum, the site of the primary infection, was increased in TRAIL-treated mice compared to placebo-treated mice [54]. This result suggests that neutrophil granulocytes during inflammation alter their sensitivity to TRAIL-induced apoptosis in the various organs and over time. These findings from inflammation models make clear that neutrophils apparently express TRAIL, as well its receptors, which under certain circumstances can induce apoptosis in these cells. The fact that neutrophils can release TRAIL indicates a cytotoxic anti-tumor effect of these cells. Accordingly, IFNγ-stimulated neutrophils have been shown to exert TRAIL-dependent cytotoxic effects on Jurkat cells [61].

Generally, the fact that neutrophils expand both in the TME and in the periphery of tumor patients, and that this correlates with a poor prognosis, puts neutrophils back in focus. Neutrophils, like macrophages, can be polarized into different activation states, resulting in pro- or anti-tumor functions [62]. Evidence suggests that polarization of neutrophils into a pro-angiogenic and immunosuppressive phenotype is associated with the downregulation of TRAIL [63].

Much evidence exists that TRAIL expression and TRAIL release of neutrophils are important elements of the therapeutic effect of *Mycobacterium bovis* bacillus Calmette–Guérin (BCG) in bladder carcinoma [64]. Intravesical instillation of BCG has been used for decades in bladder cancer after local resection, based on substantial evidence. BCG is taken up into the cells of the urothelium and here leads to the release of proinflammatory cytokines [64]. This is followed by a cellular response, which is initially dominated by neutrophils, while later Th1 cells also play a role. The neutrophils recruited through BCG therapy express significant amounts of TRAIL and can also release TRAIL from intracellular granules [64,65]. High levels of TRAIL in the bladder correlate with the efficacy of BCG therapy [64]. Accordingly, in vitro experiments have shown that neutrophils release TRAIL through stimulation with BCG [66]. Further publications suggest that the release of TRAIL occurs through activation of Toll-like receptor (TLR) 2 or TLR4 on neutrophils [66].

## 8. TRAIL and its Interactions with T cells: Lessons Learned from Autoimmunity and Infection

The increased expression of TRAIL in immune-privileged organs was the initial indication of a crucial role of TRAIL in immune tolerance that promoted the deletion of autoreactive T and B cells [67]. Through investigation of the pleiotropic effects of TRAIL on immune cells, T lymphocytes became a focus of interest early on, as mice deficient for TRAIL (TRAIL^−/−^) displayed an enhanced severity of autoimmune disease compared with their wild-type littermates [58,68,69]. While TRAIL inhibits T helper 1 (Th1) cells, it promotes the proliferation of CD4^+^ regulatory T cells (Tregs) [58]. Consequently, increased numbers of Th1 cells have been found within the spleen and intestinal lymph nodes of TRAIL^−/−^ mice. Furthermore, Ikeda et al. demonstrated that TRAIL expression on bone marrow-derived dendritic cells enhanced the proliferation of regulatory T cells [58]. The different effects of TRAIL on both T cell subtypes were accompanied by variable expression of TRAIL receptors: both cell types expressed DR5, but only Tregs were additionally characterized by decoy receptor mDcR1 (murine Decoy receptor 1) expression. TRAIL has also been proposed to play a crucial role in the suppressive properties of Tregs [70].

In contrast, Th2 cells were obviously TRAIL-resistant, and Th17 cells were not influenced in their proliferative capacity by TRAIL [71]. Park et al. showed decreased IL-17 levels after TRAIL therapy due to the induction of apoptosis in Th17 cells in a model of experimental inflammatory arthritis [72].

The inhibitory effects of TRAIL on T cells have been clearly illustrated by showing that the combined stimulation of TRAIL with anti-CD3/anti-CD28 significantly decreased the expression of activation markers, as well as Th1 and Th2 cytokines, compared with stimulation with anti-CD3/anti-CD28 alone [73]. Furthermore, TRAIL impaired the proliferation of anti-CD3/anti-CD28–stimulated T cells without the induction of apoptosis due to the TRAIL-mediated prevention of proximal T cell receptor activation. Most importantly, TRAIL-R costimulation can inhibit alloantigen-specific T cell proliferation, and impairs the activation and proliferation of autoreactive T cells [73]. These data are in line with reports demonstrating that TRAIL expressed by dendritic cells can suppress T cell proliferation [74], and have recently been confirmed in a model of autoimmune encephalomyelitis [68].

**Figure 3:**
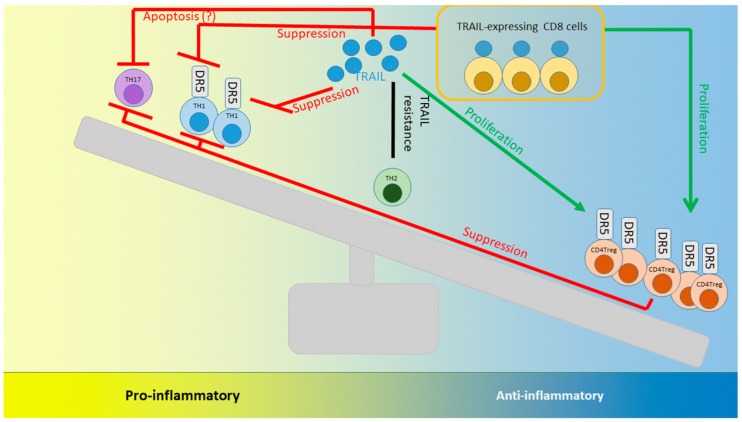
Interactions of TRAIL with T cells. While TRAIL inhibits the proliferation of Th1 cells, TRAIL expressed on dendritic cells has been shown to enhance the proliferation of CD4 regulatory cells [58]. Furthermore, a TRAIL-expressing population of CD8 cells exerting regulatory properties has been identified [56]. While Th2 cells were resistant to TRAIL-induced apoptosis in in vitro experiments, contradictory data exist in the literature for the regulation of TH17 cells [71,72].

TRAIL has been further suggested to be a key molecule in the action of regulatory CD8^+^ T cells, as it has been demonstrated that sepsis-induced apoptosis suppresses a delayed-type hypersensitivity and that this effect is dependent on TRAIL-expressing CD8^+^ T cells. This observation and other publications suggest the existence of a population of TRAIL-expressing CD8^+^ T cells with regulatory properties [56,75,76]. These cells act according to different regulatory mechanisms: they either secrete anti-inflammatory cytokines and induce the formation of tolerogenic cells, which may in turn be able to regulate the proliferation and activation of CD4^+^ T cells, or alternatively, they interact via direct cell–cell contact to suppress CD4^+^ T cells. Regulatory CD8^+^ T cells also secrete cytotoxic substances and induce cytolysis of antigen-activated CD4^+^ T cells [77]. Furthermore, the regulatory capacity of CD8^+^ Tregs has been shown to be significantly higher than that of regulatory CD4^+^ T cells in vivo [78]. Interactions of TRAIL with T cells are shown in Figure 3.

The pleiotropic effects of TRAIL on neutrophils and on T cells lead to conflicting results in peritonitis models and are a possible explanation for the sometimes seemingly contradictory results derived from infection models. Thus, TRAIL^−/−^ mice display a survival advantage in a murine model of polymicrobial sepsis, an observation that is independent from neutrophils [79]. In concordance with these results, Gurung et al. found that TRAIL^−/−^ mice were better able to control a second-hit infection following peritonitis, suggesting that sepsis-induced immunosuppression was TRAIL-dependent. This effect was attributed to interactions of TRAIL with T cells [80].

The expression of TRAIL by CD8^+^ T cells further indicates an additional role for TRAIL in viral infections. Brincks et al. found that disease severity is enhanced in TRAIL^−/−^ mice, indicating a protective role of TRAIL in influenza A virus (IAV) infection. In their study, TRAIL was mainly expressed on murine influenza–specific CD8^+^ T cells, while influenza virus–infected epithelial cells selectively expressed high amounts of DR5 [81].

Conversely, a detrimental effect of TRAIL has been illustrated in IAV-induced lung injury. IAV infection results in both the release of IFNα by alveolar epithelial cells and induction of IFNβ-dependent release of TRAIL in alveolar macrophages. Ligation to their receptors, i.e., IFNAR and particularly DR5, results in impairment of the ion channel NaK-ATPase. This leads to enhanced ion and fluid transport from the alveolar airspace toward the interstitium, resulting in extensive edema formation in the lung following IAV infection [82].

In a murine model of chronic cytomegalovirus (CMV) infection, activated antiviral CD4^+^ T cells were eliminated by TRAIL-expressing NK cells in the salivary glands, prolonging the chronicity of infection. However, this effect also constrained viral-induced autoimmunity antibodies in parallel, underlining the protective effect of TRAIL in autoimmunity [83]. Verma et al. further elucidated the importance of TRAIL in CMV infection by demonstrating that the murine CMV m166 open reading frame is required to restrict the expression of TRAIL-R2 on CMV-infected cells, and thus prevents the cell from being eliminated by the immune system, promoting early phase replication [84]. In another work, the same research group has now shown that m166 is equally important for viral persistence in the salivary gland. However, the key finding of this work was that tissue-derived ILC1s, but not conventional NK cells, showed high levels of TRAIL expression during both early and persistent infection [85].

## 9. TRAIL’s Effects on Tumor Immunology

Evidence from TRAIL^−/−^ mice showed an increased susceptibility of these animals to tumors. TRAIL^−/−^ mice significantly more often spontaneously developed lymphoid tumors in older age than wild-type animals. The susceptibility of these animals to not only lymphoid but also stromal tumors could be further increased by the loss of at least one p53 allele [86]. In the A20 B cell lymphoma transferred model, TRAIL^−/−^ mice were more susceptible to death from tumor burden [87]. Furthermore, injection of Renca cells into the spleen of TRAIL^−/−^ mice led to increased metastasis compared to wild-type mice [2]. Finnberg et al. showed in lymphoma-prone mice that lymphomas in TRAIL-R^−/−^ mice had a decreased median lymphoma-free survival [88]. In a murine squamous cell carcinoma model, TRAIL-R^−/−^ mice showed increased lymph node metastases while the development of the primary tumor was not affected [89]. Adherent TRAIL-R-expressing skin carcinoma cells were TRAIL-resistant, but were sensitized to TRAIL on detachment by inactivation of the ERK signaling pathway [89]. All these results underline the importance of TRAIL for the development and progression of tumors.

Generally, regarding the effects of TRAIL on tumor immunology, two mechanisms are conceivable: a direct effect of TRAIL on immune cells, and an indirect influence via released cytokines by tumor cells on TRAIL stimulation on immune cells. TRAIL can activate alternative signal transduction pathways, provoking the expression of NF-kB, MAPK, Src, and PI3K, and resulting in the production of proinflammatory candidate markers, including IL-8, uPA, and the matrix metalloproteinases (MMP)-7 and -9 [90]. In this context, TRAIL receptor stimulation has recently been shown to result in the activation of NF-kB and the consecutive release of proinflammatory cytokines in tumor cell lines. Although caspase activity was not required for TRAIL-induced cytokine production, caspase 8 was found to act in a nonenzymatic role as scaffolding for the assembly of a caspase 8-FADD-RIPK1 “FADDosome” signaling complex and downstream activation of NF-κB [91]. Similarly, TRAIL stimulation of pancreatic carcinoma cell lines via DR4 induced the release of uPA and IL-8 [92]. In particular, IL-8 stimulates tumor proliferation and activates endothelial cells within the tumor, resulting in an invasion of granulocytes and stimulation of tumor-associated macrophages [93], finally leading to tumor progression.

Whereas dying cells are the main source of cytokine release following FasL stimulation, viable cells that survive TRAIL stimulation have been shown to be the main source of TRAIL-induced cytokine secretion [94]. In a model of lung cancer, Hartwig et al. determined that the cytokine secretion of TRAIL-resistant cancer cells on TRAIL stimulation is dependent on FADD and leads to the polarization of monocytes to MDSCs (myeloid-derived suppressor cells) and M(IL-4)-like macrophages dependent on CCL2 (chemokine (C-C motif) ligand 2) [94].

Additionally, we have shown in a syngeneic orthotopic model of murine pancreatic cancer that TRAIL significantly provokes the immune system toward a more suppressive phenotype, thereby promoting tumor growth. The murine pancreatic carcinoma cell line 6606 PDA used for these experiments was resistant to TRAIL-induced apoptosis. Thus, TRAIL stimulation failed to induce apoptosis or cell proliferation in vitro. Applying this cell line in an orthotopic model of pancreatic cancer, TRAIL^−/−^ mice survived significantly longer and had smaller tumor volumes than wild-type controls. Additionally, while no differences were found in the lymphatic organs, TRAIL^−/−^ mice demonstrated lower frequencies of CD4^+^ Tregs in the TME [95], which could be reversed by the application of recombinant TRAIL. It remains unclear whether this effect is a result of an induced cytokine release by the tumor itself or a direct interaction of TRAIL with the cells of the immune system. As respective tumors expressed TRAIL receptors, as well as TRAIL itself to a high degree, cytokine induction after binding of TRAIL on tumor cells cannot be ruled out [95]. However, the above-presented data from inflammation models also suggest a direct effect of TRAIL on the cells of the immune system. It is therefore additionally conceivable that the direct inhibition of T cell activation and T cell proliferation, which has been described repeatedly in models of autoimmune diseases, is responsible for the shift in the T cell equilibrium towards Tregs.

## 10. Conclusions

The interactions of TRAIL with the immune system in the context of tumor diseases are complex and not yet completely understood. Its pleiotropic effects, mediated by the release of cytokines from tumor cells and/or its inhibition of the activation and proliferation of T cells, lead to a tumor-promoting TME. Consequently, these interactions of TRAIL with the immune system needs to be considered for further evaluation and development of potential indications considering TRAIL/TRAIL-R therapy.
